# Green Synthesis of Silver Nanoparticles Using *Populi gemmae* Extract: Preparation, Physicochemical Characterization, Antimicrobial Potential and In Vitro Antiproliferative Assessment

**DOI:** 10.3390/ma15145006

**Published:** 2022-07-19

**Authors:** Brigitta Kis, Elena-Alina Moacă, Lucian Barbu Tudoran, Delia Muntean, Ioana Zinuca Magyari-Pavel, Daliana Ionela Minda, Adelina Lombrea, Zorita Diaconeasa, Cristina Adriana Dehelean, Ștefania Dinu, Corina Danciu

**Affiliations:** 1 Department of Pharmacognosy, Faculty of Pharmacy, “Victor Babeș” University of Medicine and Pharmacy, 2nd Eftimie Murgu Sq., 300041 Timisoara, Romania; kis.brigitta@umft.ro (B.K.); ioanaz.pavel@umft.ro (I.Z.M.-P.); minda.daliana@umft.ro (D.I.M.); lombrea.adelina@yahoo.com (A.L.); corina.danciu@umft.ro (C.D.); 2 Research Center for Pharmaco-Toxicological Evaluation, “Victor Babeș” University of Medicine and Pharmacy Timisoara, 2nd Eftimie Murgu Sq., 300041 Timisoara, Romania; cadehelean@umft.ro; 3 Department of Toxicology and Drug Industry, Faculty of Pharmacy, “Victor Babeș” University of Medicine and Pharmacy, 2nd Eftimie Murgu Sq., 300041 Timisoara, Romania; 4 Electron Microscopy Laboratory “Prof. C. Craciun”, Faculty of Biology & Geology, “Babes-Bolyai” University, 5-7 Clinicilor Street, 400006 Cluj-Napoca, Romania; lucianbarbu@yahoo.com; 5 Electron Microscopy Integrated Laboratory, National Institute for R & D of Isotopic and Molecular Technologies, 67-103 Donat Street, 400293 Cluj-Napoca, Romania; 6 Department of Microbiology Faculty of Medicine “Victor Babeș” University of Medicine and Pharmacy, 2nd Eftimie Murgu Sq., 300041 Timisoara, Romania; muntean.delia@umft.ro; 7 Department of Food Science and Technology, Faculty of Food Science and Technology, University of Agricultural Science and Veterinary Medicine, 3-5 Calea Manastur, 400372 Cluj-Napoca, Romania; zorita.diaconeasa@gmail.com; 8 Department of Pedodontics, Faculty of Dental Medicine, “Victor Babeș” University of Medicine and Pharmacy, 9 Revolutiei Bulevard, 300041 Timișoara, Romania; dinu.stefania@umft.ro; 9 Pediatric Dentistry Research Center, Faculty of Dental Medicine, “Victor Babeș” University of Medicine and Pharmacy, 9 Revolutiei Bulevard, 300041 Timișoara, Romania

**Keywords:** green synthesis, *Populus nigra* L., silver nanoparticles, antimicrobial potential, antiproliferative activity

## Abstract

Green route is an economic, facile and eco-friendly method, employed for the synthesis of various types of nanoparticles, having it as a starting point biological entity, especially as a plant extract. The present study aims to obtain silver nanoparticles (AgNPs) starting from an ethanolic extract of *Populi gemmae* (Pg), by adjusting the reaction parameters. The morphological and structural characterization exhibited that both the reaction temperature and the concentration of metal salt, contributes to the obtaining of Pg-AgNPs with adjustable size and shape. The newly synthesized nanoparticles exhibited a good antibacterial activity on Gram-positive bacteria as well as antifungal activity. The in vitro antiproliferative activity of Pg-AgNPs was assessed on two different cancer cell lines (breast cancer cells—MCF7 and lung carcinoma epithelial cells—A549). Results have shown that the green-synthetized Pg-AgNPs_S2 (obtained at 60 °C, using AgNO_3_ of 5 M) induced a substantial decrease in tumor cell viability in a dose-dependent manner with an IC_50_ ranging from 5.03 to 5.07 µg/mL on A549 cell line and 3.24 to 4.93 µg/mL on MCF7 cell line.

## 1. Introduction

Nowadays, nanotechnology is receiving more and more interest as a field of study dealing with the production of nanomaterials, useful in a variety of areas including biomedicine, drug-delivery, bioimaging, pharmaceutic, optoelectronics, catalysis, bio-sensing devices, food technology and cosmetology, due to their high biocompatibility, rapid productivity and cost-effectiveness [1,2,3,4,5,6,7,8,9]. Nanotechnology is considered an important tool in the production of materials with interatomic structural features. Nanoparticles (NPs) are atomic or molecular scale solid materials that exhibit superior physical characteristics in comparison to bulk materials, relying on their size and shape [10]. Owing to their tailorable physicochemical properties, NPs may be used in the pharmaceutical industry to improve drug delivery and regulate drug release [11]. Especially, NPs of gold (Gd), silver (Ag) or platinum (Pt) (noble metals), are recognized to gain popularity due to their outstanding properties and versatility [12]. When compared to larger particles with the same chemical composition, AgNPs possess a significant surface area, which results in notable activity (biological and/or catalytic), and atomic behavior [13]. Numerous techniques for the generation of NPs have been documented, including chemical (precipitation, reduction, sol-gel or polyol synthesis) and physical approaches (microwave-assisted combustion, laser pyrolysis or laser evaporation) [14,15]. Although their widespread usage, chemical and physical procedures have multiple disadvantages, such as: the use of toxic raw materials, the emanation of hazardous secondary products, increased production cost and limited yield [14]. To address the aforementioned issues, the approach of green bio-synthesis of NPs is regarded as state of the art within the nanotechnology field [16]. Biological synthesis referred to as green synthesis, outperforms conventional synthesis methods by being a simple, cheap, viable and biocompatible technique. It is regarded as environmentally and ecologically sustainable since it makes use of readily accessible resources such as fungus, bacteria, algae or plant extracts that serve as reducing and/or capping/stabilizing agents [17]. Moreover, it’s also simple to scale up, employs nontoxic solvents and is devoid of unwanted by-products [14,15,18,19,20].

The Plant Kingdom is well recognized for producing plant-derived NPs due to the large number of phytocompounds contained in plant extracts, such as aldehydes, ketones, amides, flavonoids, carboxylic acids, phenols, terpenoids and ascorbic acids [21]. Up to this point, NPs have been synthesized using a wide variety of plant extracts (e.g., *Ocimum sanctum* L., *Ginkgo biloba* L., *Equisetum arvense* L. leaves extract, *Salvia hispanica* L. seed extract; *Bauhinia acuminate* L., *Datura inoxia* Mill. flower extract, etc.) [15,22,23,24,25,26,27]. Plant-based techniques include combining a plant extract with the aqueous solution of a metal salt. This procedure occurs at room temperature, and might take anything from a few minutes to a few hours to finish [28].

Green chemistry techniques for AgNPs synthesis have recently advanced, demonstrating promising activity in the medical field due to their anticancer, anti-inflammatory, anti-diabetic, antimicrobial, anti-angiogenic, wound-healing and anti-coagulating properties [29,30]. For instance, the group of Sankar et al. [31], has obtained AgNPs using *Origanum vulgare* L. aqueous extract, which has been shown to possess antibacterial and anticancer activities in a dose-dependent manner. Moreover, AgNPs produced from *Salvia officinalis* L. extract have been shown to possess an effective anti-angiogenic potential when applied to chick chorioallantoic membrane (CAM) by decreasing blood hemoglobin levels [32]. Additionally, plant-based AgNPs have emerged as strong antibacterial agents owing to their efficiency, which exceeds that of commonly used antibiotics. For instance, Abalkhil et al. [33] synthesized AgNPs from *Aloe vera* L., *Portulaca oleracea* L. and *Cynodon dactylon* L. aqueous extracts and assessed their antibacterial activity towards Gram-positive and Gram-negative human pathogenic bacteria. The research group demonstrated that cell wall destruction was the primary event occurring during the antibacterial activity of AgNPs. Moldovan et al. [34] used *Viburnum opulus* L. fruit extract to produce AgNPs. They have shown an in vitro anti-inflammatory effect against the human epidermal keratinocyte (HaCaT) cell line. The synthesized AgNPs from *Viburnum opulus* L. exhibited an inhibitory effect against cyclooxygenase (COX), a key mediator of inflammation.

*Populus nigra* L. (*Populi gemmae*), popularly known as the black poplar tree, is a resourceful participant of the *Salicaceae* family. The *Populus* genus is made up of about 40 species that are found across the world, particularly in Europe and Asia [35]. Different types of extracts obtained from leaves and bark of *Populus nigra* L. may all be employed as active components in pharmaceuticals, although in recent years, the resinous buds have been in the spotlight of numerous research studies [36,37,38]. Due to their extensive phytochemical profile, black poplar buds have been used in ethnopharmacology to treat several diseases namely, bronchitis, cough, tracheas, laryngitis, sore throat, ulcers, hemorrhoids, anal fissures, rheumatism, etc. This vegetal product has been endowed with anti-inflammatory, antipyretic, analgesic, antiallergic, antimicrobial, expectorant and capillary-protective properties [39,40,41]. Starting from these foundational shreds of evidence, an ever-growing number of research studies have portrayed novel pharmaceutical applications of black poplar buds serving as an antioxidant, antibacterial, antifungal, anti-inflammatory, antidiabetic, anticancer, hepatoprotective and hypouricemic agents [38,42,43,44,45,46].

In this context, the goal of the current study consists of the preparation through green synthesis and structural characterization of two types of Ag nanoparticles, starting from an ethanolic extract of *Populi gemmae*, as well as the preliminary assessment of the antibacterial and antiproliferative (on two different cancer cell lines—breast cancer cells (MCF7) and lung carcinoma epithelial cells (A549) potential.

## 2. Experimental Part

### 2.1. Populi Gemmae Extract Preparation

*Populi gemmae* (Pg) were harvested from the western part of Romania (Timișoara, coordinates: 45°44′58″ N latitude, 21°13′38″ W longitude) and identified at the Faculty of Pharmacy, “Victor Babeș” University of Medicine and Pharmacy Timisoara (voucher specimen code Pg 3/2019). The extraction methodology was extensively detailed in a previous study, conducted by our research team [38]. First, 100 mL of 70% ethanol was added to 10 g of dried and ground plant material and left to soak for 10 min. at 24 °C. For a more efficient extraction, the mixture was introduced for 30 min. in the ultrasonic water bath (at 50 °C) (FALC LBS 2, Treviglio, Italy). Filtration was performed using a vacuum pump (Vacuubrand) through filter paper (Whatman no. 4). After sonication, the ethanolic mixture was concentrated in a rotary evaporator (HEIDOLPH Laborata 4000 efficient WB eco, Schwabach, Germany), at 50 °C and reduced pressure. The solid extract obtained was placed in an oven (Genlab N40c, Widnes, England) for several hours at the same temperature, in order to achieve better drying. Finally, 2.300 g of Pg extract was obtained. The sample was deposited in the freezer at −4 °C until use [47].

### 2.2. Green Synthesis of AgNPs from Populi gemmae Extract

In order to prepare AgNPs by green synthesis, the slightly modified protocol of Ruiz-Baltazar et al. was employed [48]. Aliquots of Pg extract (10 mg/mL) were dispersed in 70% ethanol and subjected to a magnetic stirrer, using a magnetic bar. Over the first sample (S1) at 25 °C and 250 rpm was added an aqueous solution of 1 M AgNO_3_ in a thin thread and the whole mixture was left for 2 h at magnetic stirring. Over the second sample (S2) at 60 °C and 500 rpm was added an aqueous solution of 5 M of AgNO_3_ in a thin thread, following the mixture to stand for 2 h at thermo-magnetic stirring. The volume ratio of AgNO_3_:Pg extract was 1:2 in both cases. After 2 h, the color change of the post-reaction mixtures occurred, from light brown to black-brown, which confirmed the reduction of AgNO_3_ to AgNPs. The synthesized Pg-AgNPs_S1 (obtained at 25 °C) and Pg-AgNPs_S2 (obtained at 60 °C) starting from Pg ethanolic extract, were separated by centrifugation 6000 rpm for 30 min, and dried at 40 °C, using an oven (POL-EKO Aparatura, Wodzisław Slaski, Poland). Both formed Pg-AgNPs were further physicochemical analyzed.

### 2.3. Physicochemical Characterization of Pg-AgNPs

After biosynthesis, both types of nanoparticles were subjected to thermal analysis in order to assess the stability of phytocompounds from Pg extract and of pre-formed Pg-AgNPs. The thermal behavior of samples was studied using a Netzsch STA 449 C instrument (Netzsch-Gerätebau GmbH, Selb, Germany), in the range of 10–1000 °C and a 20 mL/min flow rate of air atmosphere. To record the thermogravimetric (TG, Netzsch-Gerätebau GmbH, Selb, Germany), derivative thermogravimetric analysis (DTA, Netzsch-Gerätebau GmbH, Selb, Germany) and differential scanning calorimetry (DSC, Netzsch-Gerätebau GmbH, Selb, Germany) curves, aluminum crucibles were used.

The functional molecules who participated in the formation of Pg-AgNPs were recorded by Fourier-transform infrared spectroscopy (FT-IR), using a Shimadzu Prestige-21 spectrometer (Shimadzu Europa Gmbh, Duisburg, Germany), at 24 °C. The FT-IR spectrometer operated at a resolution of 4 cm^−^^1^ within the range of 400–4000 cm^−^^1^, using KBr pellets. The interpretation of the bands revealed by FT-IR spectra was accomplished in accordance with the Characteristic IR Absorption Frequencies of Organic Functional Groups [49].

The aspects regarding morphology and ultrastructure of Pg-AgNPs were determined by scanning electron microscopy (SEM), using a Hitachi SU8230 cold field emission gun STEM (Chiyoda, Tokyo, Japan) microscope with EDX detectors X-Max^N^ 80 from Oxford Instruments (Abingdon, UK), in high-vacuum mode (HV) and acceleration voltage 200 kV. The identified chemical species, expressed in atomic percent (At%), were assessed by EDX. The particle sizes of Pg-AgNPs were evaluated by transmission electron microscopy (TEM, Chiyoda, Tokyo, Japan), using a Hitachi HD2700 cold field emission gun STEM (Chiyoda, Tokyo, Japan) equipped with two windowless EDX detectors (X-Max^N^ 100).

### 2.4. Antimicrobial Activity Tests

The antimicrobial activity for tested compounds was initially assessed by disk diffusion method followed by dilution method [38,50,51,52]. The selected strains are the following: *Streptococcus pyogenes* (ATCC 19615), *Staphylococcus aureus* (ATCC 25923), *Escherichia coli* (ATCC 25922), *Pseudomonas aeruginosa* (ATCC 27853), *Candida albicans* (ATCC 10231) and *Candida parapsilosis* (ATCC 22019).

#### 2.4.1. Disk-Diffusion Method

The microbial suspensions were prepared to a concentration of 0.5 McFarland, then 0.1 mL of each suspension were inoculated on Mueller–Hinton agar plates (bioMérieux, Marcy-l’Étoile, France), supplemented with sheep blood (for *Streptococcus strain*) or methylene blue and glucose (for *Candida species*). Three blank disks (BioMaxima, Lublin, Poland) impregnated with 0.01 mL of each compound were added on the top of the agar inoculated, central. The plates were incubated at 35 ± 2 °C, for 24 h. The sensitivity was considered for a diameter larger than 15 mm.

#### 2.4.2. Determination of the Minimum Inhibitory Concentrations (MIC) and the Minimum Bactericidal Concentrations (MBC) or Minimum Fungicidal Concentrations (MFC)

The MIC was determined by dilution method in Mueller–Hinton broth (supplemented according to species) using a final microbial suspension of 500,000 µorganisms/mL and final serial dilutions of compounds from 50 to 3.125 mg/mL. After 24 h of incubation, MIC was considered the lowest concentration without visible growth. The MBC/MFC were determined only from the test tubes that showed no bacterial growth in the dilution method. From these test tubes, 0.001 mL was inoculated on Columbia agar +5% sheep blood or Sabouraud and the plates were incubated for 24 h at 35 ± 2 °C. The MBC or MFC was considered for the concentration which killed 99.9% of the microorganisms.

### 2.5. Cell Culture

The lung adenocarcinoma cell line (A549, ATCC^®^ CCL-185TM) and human breast adenocarcinoma (MCF7, ATCC^®^ HTB-22TM) were purchased from the American Type Culture Collection (ATCC). The A549 cells were cultured in Dulbecco’s Modified Eagle’s Medium (DMEM; Sigma-Aldrich, Darmstadt, Germany) and the MCF7 cells in RPMI-1640 medium (ATCC^®^ 30-2001™). Both cell lines were supplemented with 10% fetal bovine serum (FBS; Sigma-Aldrich, Taufkirchen, Germany) and 1% penicillin/streptomycin mixture (P/S, 10,000 IU/mL; Sigma-Aldrich, Darmstadt, Germany) and further preserved at 37 °C and humidified atmosphere with 5% CO_2_.

### 2.6. MTT Assay

The selected concentrations of Pg-AgNPs were evaluated for their in vitro anticancer activity against A549 and MCF7 cell lines, using MTT (3-(4,5-dimethylthiazol-2-yl)-2,5-diphenyltetrazolium bromide) assay [53]. Approximately 1 × 10^5^ cells/well from each cell line were seeded in a flat-bottomed 96 well plate and allowed to adhere overnight at 37 °C in a 5% CO_2_ incubator. After incubation, the cells were stimulated with the selected concentrations (10, 25, 50, 75, 100 and 150 µg/mL) of Pg-AgNPs (in the medium). After 24 and 72 h of incubation, respectively, the cells were treated with 10 µL of 5 mg/mL MTT solution (Sigma-Aldrich, St. Louis, MO, USA) and were incubated for another 3 h. After this time, 100 µL of lysis solution (from the MTT kit) was added to each well in order to obtain the formazan crystals. Samples were left at room temperature for additional 30 min. The absorbance was read at 570 nm, using a microplate reader (Tecan, Spectrophotometer, Durham, NC, USA) 24 and 72 h post exposure. Control cells were considered the untreated cells (cells treated only with cell culture medium).

### 2.7. Statistical Analysis

GraphPad Prism 5.0 Software (GraphPad Software, San Diego, CA, USA) and Origin 2020b (Origin Lab—Data Analysis and Graphing Software, Origin 2020b, Szeged, Hungary) were the statistical software used in the current study. The in vitro experiments were carried out in quadruplicate. The results were expressed as a mean ± standard deviation (SD). For the MTT assays, the statistical differences were performed using the One-way ANOVA test followed by Tukey’s test. A *p*-value of ≤0.05 was considered to be of statistical significance (* *p* ≤ 0.05; ** *p* ≤ 0.01; **** *p* ≤ 0.0001). The IC_50_ values were determined by employing AAT Bioquest’s online IC_50_ calculator [54].

## 3. Results

### 3.1. Physicochemical Screening of Pg-AgNPs

#### 3.1.1. Thermal Behavior

**Figure 1:**
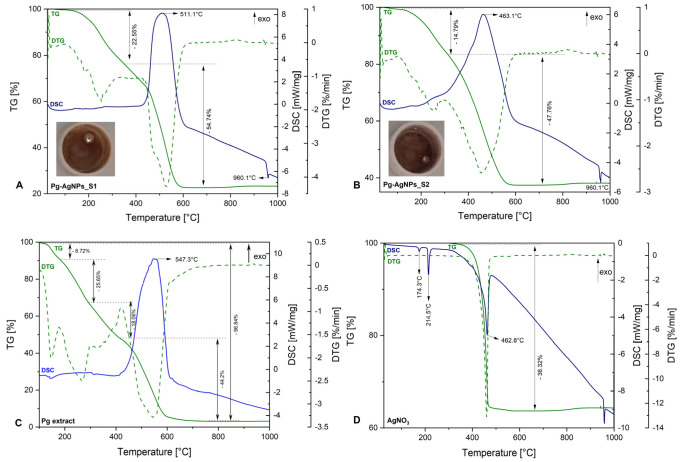
TG—DSC curves: (**A**)—Pg-AgNPs_S1 curves from Pg extract at 25 °C by green synthesis; (**B**)—Pg-AgNPs_S2 curves from Pg extract at 60 °C by green synthesis; (**C**)—Pg ethanolic dried extract; (**D**)—AgNO_3_ curves.

Figure 1 shows the TG-DSC curves of both types of Pg-AgNPs obtained by green synthesis, as well as the TG-DSC curves of AgNO_3_ and Pg ethanolic extract.

Regarding the thermal behavior of the synthesized Pg-AgNPs (Figure 1A,B), it can be observed that independent of reaction conditions, both graphics are very similar. Both samples recorded a mass loss in two stages, but, the second stage of mass loss, is accompanied with an exothermic effect, recorded on a DSC curve.

The first mass loss in both samples, highlighted on the TG curves, which has no effect on the DSC curve, is due to the water elimination from the samples. In the case of the sample Pg-AgNPs_S1, in which the AgNPs were obtained at 25 °C, an exothermic effect with a maximum of 511.1 °C was recorded, with a mass loss of 54.74% on a TG curve. Meanwhile, in sample Pg-AgNPs_S2, in which the AgNPs were obtained at 60 °C, the exothermic effect on a DSC curve was at 463.1 °C, with 47.76% mass loss noticed on a TG curve. These processes are assigned to the degradation of aromatic compounds, carbohydrates, conjugated acids, alkenes, aromatic esters and aromatic amino acids present at the surface of AgNPs, coming from the extract of Pg, as well as to nitrogen compounds from AgNPs.

In Figure 1C is, depicted. the TG-DSC curves of Pg ethanolic dried extract, extensively explained in our previous study [38]. It can be observed that the thermal behavior of Pg extract is similar with those of Pg-AgNPs, in the temperature range of 400–800 °C, when the Pg extract complete degradation take place due to the largest mass loss recorded on a TG curve (44.2%) alongside with the exothermic effect (547.3 °C), recorded on a DSC curve. After 800 °C no mass loss or exothermic process were noticed.

Regarding the thermal behavior of AgNO_3_ (Figure 1D), it can be observed an endothermic effect at 174.3 °C on a DSC curve, without mass change on a TG curve. This process corresponds to the polymorphic transformation of AgNO_3_. Another endothermic effect recorded with mass change can be observed at 214.5 °C on a DSC curve, which corresponds to solid AgNO_3_ melting to liquid AgNO_3_. The last endothermic effect noticed on a DSC curve, recorded at 462.8 °C, with a mass loss of 36.32% highlighted on a TG curve, corresponds to the decomposition of AgNO_3_ in metallic Ag and nitrogen oxides. Regarding all three graphics (Figure 1A,B,D), at around 960 °C can be observed an endothermic effect, without mass loss on TG curves, in which take place the metallic Ag melting, a process noticed also in the photographs overlapped on the first two graphics (Figure 1A,B).

#### 3.1.2. FT-IR Investigations

**Figure 2:**
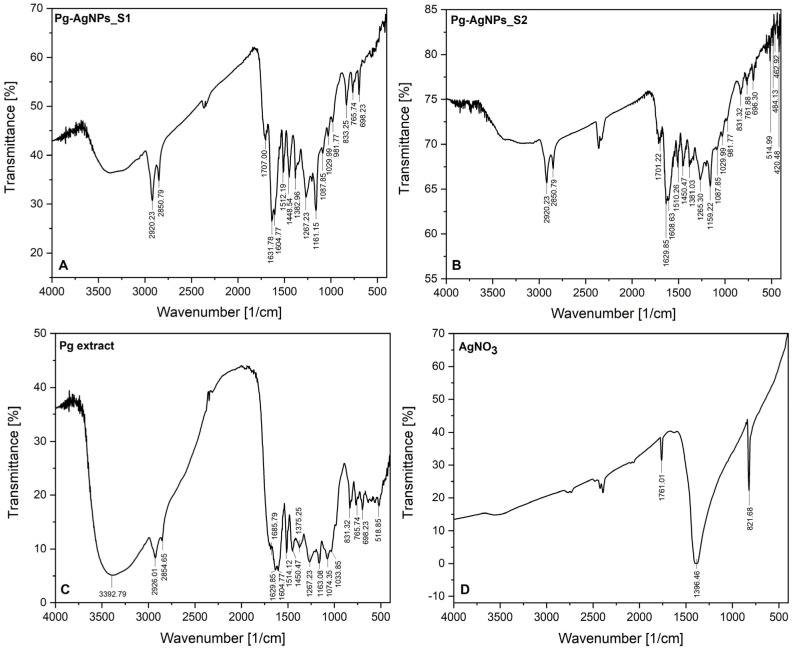
FT—IR spectra: (**A**)—Pg-AgNPs_S1 spectrum from Pg extract, obtained at 25 °C by green synthesis; (**B**)—Pg-AgNPs_S2 spectrum from Pg extract, obtained at 60 °C by green synthesis; (**C**)—Pg ethanolic dried extract; (**D**)—AgNO_3_ spectrum.

The FT-IR analysis was performed in order to determine the functional molecules of *Populi gemmae* ethanolic extract which act as capping and reducing agents, being involved in the green synthesis of AgNPs. The evidence of the phytocompounds associated with the *Populi gemmae* showed by FT-IR spectra AgNPs obtained, is presented in Figure 2. As showed in Figure 2, the FT-IR spectrum of Pg-AgNPs_S1 (Figure 2A) was similar to the FT-IR spectrum of Pg-AgNPs_S2 (Figure 2B). For comparison, we attached the FT-IR spectrum of the Pg ethanolic dried extract (Figure 2C), explained in detail in a previous study published by our research team [38].

From the FT-IR spectra of both Pg-AgNPs (Figure 2A,B), it can be seen that, the peaks recorded at 2920.23 and 2850.79 cm^−1^, corresponds to the C-H stretching vibration of saturated aliphatic groups from Pg extract (recorded at 2926.01 and 2854.65 cm^−1^—Figure 2C). The band around 1700 cm^−1^ corresponds to C=O stretching vibration of conjugated acids formed at the surface of Pg-AgNPs, observed at 1685.79 cm^−1^ on Pg extract FT-IR spectrum. It can be observed in the AgNO_3_ spectrum (Figure 2D) a band located at 1761.01 cm^−1^, due to the conjugation of acids, the absorption peaks are moved to a lower wavenumber (1707.00 cm^−1^ in the case of Pg-AgNPs_S1 and 1701.22 cm^−1^ in the case of Pg-AgNPs_S2). The peaks recorded around 1600 cm^−1^ are assigned to the C=C stretching vibration from aromatic compounds contained in Pg extract, due to the multiple bands recorded and the medium-weak intensity of the peaks. The peaks around 1500 cm^−1^, as well as the peaks around 1300 cm^−1^, are attributed to the N-O stretching vibration; these peaks demonstrate that the AgNPs were synthesized. The medium intense bands recorded around 1400 cm^−1^ on both Pg-AgNPs spectra (Figure 2A,B) are associated with the C-N stretching vibration of the aromatic amine groups. The aromatic amine groups are evidenced on Pg extract FT-IR spectrum at 1267.23 as well as 1163.08 cm^−1^ (Figure 2C). The absorbance peaks evidenced by FT-IR on both spectra, located between 1300–1000 cm^−1^, indicate the residual moieties of the phytocompounds present in Pg extract, which are found on the surface of the formed Pg-AgNPs, namely carbonyl acids (C-O stretching vibration bands); phenolic acids (C-N bands from aromatic amines); monoterpenes and non-terpenes (C-O stretching vibration bands from alcohol function groups); flavonoids/flavonols (C-O stretching vibration bands from aromatic esters functional groups). The rest of the absorbance peaks located between 400–1000 cm^−1^, on both Pg-AgNPs spectra (Figure 2A,B), are assigned to alkene functional groups (bending vibration of =C-H bands from aldehydes and/or to the stretching vibration of C-Cl/C-Br bands from halo compounds). These peaks are also noticed on Pg extract FT-IR spectrum (Figure 2C).

On both spectra of Pg-AgNPs, can be appreciated the presence of several peaks at 698.23 cm^−1^ vs. 696.30 cm^−1^; 833.25 cm^−1^ vs. 831.32 cm^−1^; 765.74 cm^−1^ vs. 761.88 cm^−1^, which corresponds to the stretching vibration of N-O in plane from (NO_3_)^−1^ ion. In addition, on FT-IR spectrum of AgNO_3_ (Figure 2D), it can be observed a peak at 821.68 cm^−1^. This result represents another confirmation that the AgNPs in both samples were synthesized.

#### 3.1.3. Electron Microscopy Analysis

**Figure 3:**
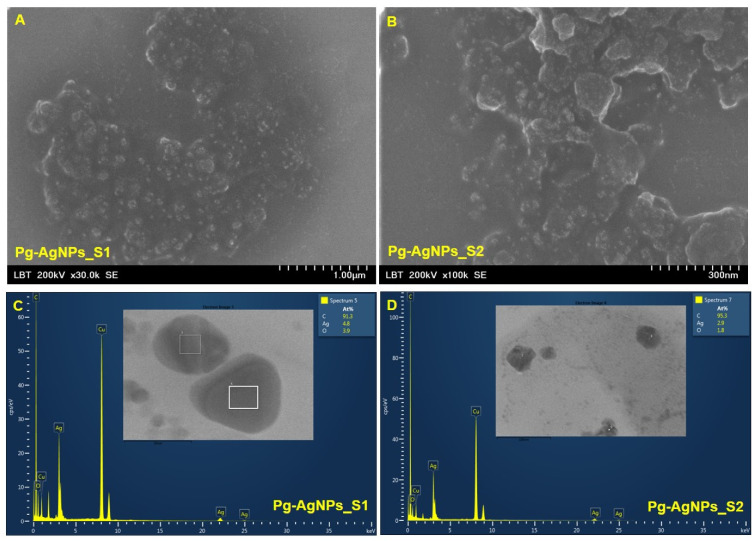
SEM-EDX images of Pg-AgNPs obtained by green synthesis, at 25 °C (Pg-AgNPs_S1—**A** and **C**) and at 60 °C (Pg-AgNPs_S2—**B** and **D**).

Figure 3A,B shows representative SEM images of Pg-AgNPs obtained by green synthesis at 25 °C and 60 °C and Figure 3C,D shows the chemical composition of Pg-AgNPs obtained by green synthesis.

**Figure 4:**
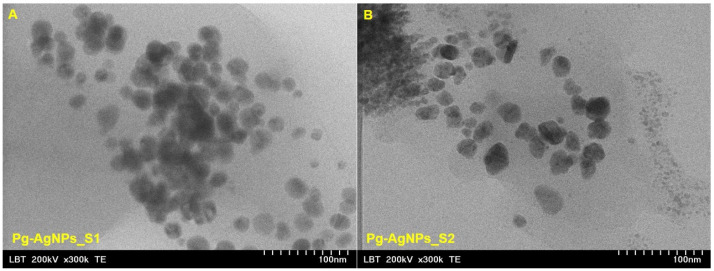
TEM images of Pg-AgNPs obtained by green-synthesis, starting from Pg extract, at 25 °C (**A**—Pg-AgNPS_S1) and at 60 °C (**B**—Pg-AgNPs_S2).

The nanoparticles were carefully placed on a glass coverslip and let to be air-dried, followed by a sputter-coated with carbon using a coater (Agar Automatic Sputtercoater, Essex, UK). Different shapes of AgNPs were obtained due to the different temperatures set in the synthesis process as well as the different concentrations of an aqueous solution of AgNO_3_ (25 °C and 1 M for the obtaining of Pg-AgNPs_S1 vs. 60 °C and 5 M for the obtaining of Pg-AgNPs_S2). The Pg-AgNPs_S1 (Figure 3A) obtained at 25 °C by using 1 M AgNO_3_, are spherical or cvasi-spherical compared to the NPs obtained at 60 °C with 5 M AgNO_3_—which seems to have an irregular shape (rhombohedral, triangular and spherical). A similar trend is also observed in the TEM images (Figure 4A,B).

The EDX profile recorded from the Pg-AgNPs shows microelements present in the sample (C, Ag, O and Cu), identified by the peak amplitude, from where it is observed that the silver signal is stronger than oxygen. The strong carbon and copper signals obtained can come from the phytocompounds bound to the surface of Pg-AgNPs, but the presence of carbon signal is also due to the carbon sputter-coated for better conductivity. The EDX spectra revealed the presence of peak amplitudes of silver at approximately 3 keV, 22 keV and 25 keV.

The TEM images of Pg-AgNPs (Figure 4) obtained by green synthesis, points out the pre-formed AgNPs starting from Pg extract. In the case of Pg-AgNPs_S1 obtained at 25 °C, it can be observed that the NPs are polydisperse, spherical or cvasi-spherical with the particle size distribution between 3 to 60 nm (Figure 4A). Regarding the Pg-AgNPs_S2, obtained at 60 °C (Figure 4B), these are also polydisperse with irregular shape and particle size distribution between 5 to 150 nm.

### 3.2. Antimicrobial Activity

Another purpose of this study was to analyze the antimicrobial activity of the selected nanoparticles (Pg-AgNPs_S1 and Pg-AgNPs_S2), which are shown in Table 1 and Table 2. It can be seen that both Pg-AgNPs presented an antibacterial activity on Gram-positive bacteria as well as on fungi. Lack of activity can be seen for Pg-AgNPs_S1 on Gram-negative bacteria. Based on the different wall structures of the respective microorganisms, this can be elucidated by the fact that the Gram-negative bacteria have a complex membrane structure (the outer membrane is non-existent in Gram-positive bacteria and fungi), therefore the compounds may penetrate only through its porins (if the compounds are large molecules they can no longer cross the membrane through the porins, as a result, they become inactive).

The difference in the antibacterial activity between Pg-AgNPs-S1 and Pg-AgNPs-S2 is attributed to the particle size of Pg-AgNPs. Pg-AgNPs-S1 was synthesized using 1 M AgNO_3_, resulting in nanoparticles with smaller dimensions than Pg-AgNPs-S2, which were designed using 5 M AgNO_3_. The antimicrobial effect of Pg-AgNPs on Gram-negative bacteria can be explained by the fact that silver nanoparticles are able to bind the lipopolysaccharides on the surface of the external membrane, thereby inducing a delayed bactericidal response compared to the Gram-positive bacteria or Candida spp., where Pg-AgNPs penetrate directly into the internal structures of bacteria/fungus.

### 3.3. Antiproliferative MTT Assay

**Figure 5:**
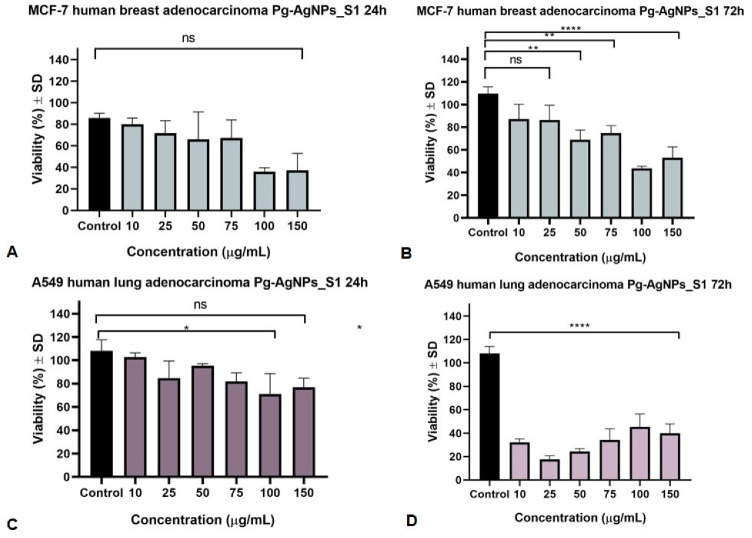
MCF7 and A549 cells viability after 24 and 72 h stimulation with Pg-AgNPs_S1 (10, 25, 50, 75, 100 and 150 μg/mL), (**A**)—24 h treatment of MCF7 cells; (**B**)—72 h treatment of MCF7 cells; (**C**)—24 h treatment of A549 cells; (**D**)—72 h treatment of A549 cells. The results are expressed as cell viability percentage (%) related to the Control cells. Comparison among groups was made using the One-way ANOVA test followed by Tukey’s test. A *p* value of ≤0.05 was considered to be of statistical significance (* *p* ≤ 0.05; ** *p* ≤ 0.01; **** *p* ≤ 0.0001).

The outcomes of the present in vitro cytotoxicity study against the selected cancer cell lines (MCF7 and A549) disclosed that Pg-AgNPs can act as a potential candidate in the management of these two types of cancer. The control group was considered to be the cells treated only with cell culture medium. Figure 5 presents the activity of Pg-AgNPs_S1 (obtained at 25 °C, using AgNO_3_ of 1 M) against MCF7 and A549 cancer cell lines after a stimulation period of 24 and 72 h. It can be seen that 72 h of incubation with Pg-AgNPs_S1 provoked a dose-dependent decrease in cell viability in both cancer cell lines, however, the most affected was the A549 cell line.

The IC_50_ values for Pg-AgNPs_S1 were 40.23 µg/mL and 48.92 µg/mL (for 24 and 72 h, respectively, of incubation of MCF7 cell line) as against 16.14 µg/mL and 4.39 µg/mL (for 24 and 72 h, respectively, of incubation of A549 cell line). One can be seen that the A549 lung cancer cell line was more sensitive to Pg-AgNPs_S1 than the MCF7 breast cancer cell line.

**Figure 6:**
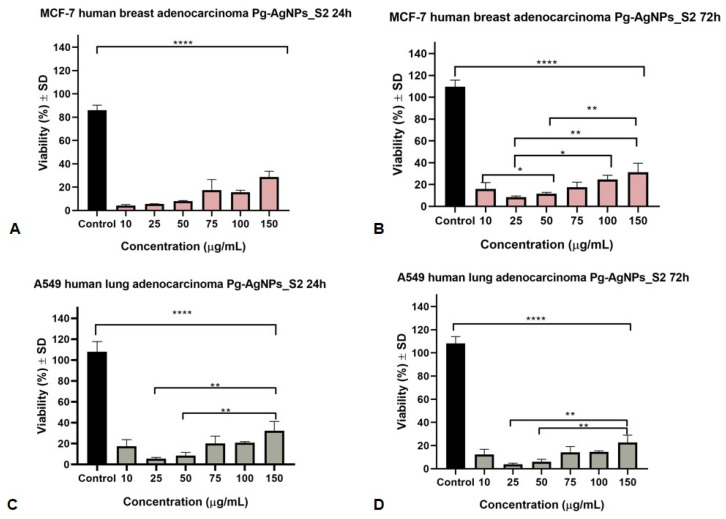
MCF7 and A549 cells viability after 24 and 72 h stimulation with Pg-AgNPs_S2 (10, 25, 50, 75, 100 and 150 μg/mL). (**A**)—24 h treatment of MCF7 cells; (**B**)—72 h treatment of MCF7 cells; (**C**)—24 h treatment of A549 cells, (**D**)—72 h treatment of A549 cells. The results are expressed as cell viability percentage (%) related to the Control cells. Comparison among groups was made using the one-way ANOVA test followed by Tukey’s test. A *p*-value of ≤0.05 was considered to be of statistical significance (* *p* ≤ 0.05; ** *p* ≤ 0.01; **** *p* ≤ 0.0001).

Figure 6 presents the activity of Pg-AgNPs_S2 (obtained at 60 °C, using AgNO_3_ of 5 M) against the two selected cancer cell lines after a stimulation period of 24 and 72 h. The IC_50_ values for Pg-AgNPs_S2 were 3.24 µg/mL and 4.93 µg/mL (for 24 and 72 h, respectively, of incubation of MCF7 cell line) in comparison to 5.03 µg/mL and 5.07 µg/mL (for 24 and 72 h, respectively, of incubation of A549 cell line). It can be noticed that the MCF7 breast cancer cell line is more sensitive to Pg-AgNPs_S2 when compared to the A549 cell line. These results indicated that an increased incubation period may be associated with significantly better results.

In terms of the antiproliferative IC_50_ values, the present study shows that Pg-AgNPs_S2 were more active on both studied cell lines. Statistically significant values appeared from 3.24 μg/mL (24 h incubation time of MCF7 cells) and 5.03 μg/mL (24 h incubation time of A549 cells). Pg-AgNPs_S1 showed a much weaker antiproliferative activity with higher IC_50_ values, the statistically significant values can be observed from 4.39 μg/mL (72 h incubation time of A549 cells) and 40.23 μg/mL (24 h incubation time of MCF7 cells).

## 4. Discussion

Nowadays, nanotechnology plays a major role in modern medicine, offering a wide range of benefits for treating human diseases through target-oriented delivery of different compounds. Nanomaterials can be defined as a material with different sizes ranging from 1 up to 100 nm that can exhibit unique chemical and biological properties [55]. Nowadays, among the major metallic-based nanoparticles utilized can be enumerated palladium, iridium, osmium, rhodium, copper, platinum, silver and gold nanoparticles [56]. It has been identified that gold and silver nanoparticles are the most commonly used in various fields of biomedical science because of their long-term stability and particular biocompatibility [57]. The AgNPs present tailorable physical and chemical properties such as size, morphology and stability. Their synthesis method plays an essential role in the preparation of less toxic AgNPs. Throughout time, the chemical and physical methods have exhibited some disadvantages (abundant energy requirement, high temperature and pressure, toxic solvents, harmful by-products, high cost and so on) toward the green method [58]. Green synthesis is a friendly method to the environment, in which plant phytocompounds act as capping, reducing and stabilizer agents, to controlling the size and prevent agglomeration of the resulted biocompatible nanoparticles. In addition, is a rapid, economical, easy and eco-friendliness method that employs natural ingredients, which are safe and less harmful to humans and nature [59]. Several parameters must be taken into account for the green synthesis of AgNPs such as: temperature, reaction time between plant extract and metal salt, pH, metal salt and plant extract concentrations. All of these factors are crucial to determine the quality, yields, morphology, size and shape of AgNPs. Characterization techniques are a crucial part for the analysis on the new synthetized nanoparticles in order to confirm the formation, and to define their structure and composition as well.

On this matter, the current study presents the preparation of silver NPs through the green route, using a volume ratio of 1:2 AgNO_3_ to plant extract, at two different temperatures (25 °C and 60 °C). The synthesized AgNPs were surface coated with biomolecules from plant extract (*Populi gemmae*), which makes them biocompatible for medical applications. A physicochemical screening of the obtained AgNPs was performed, such as TG-DSC analysis, FT-IR investigation and electron microscopy investigations (TEM and SEM-EDX), in order to confirm the stability, the functional groups attached on NPs surface, as well as the size and shape of the newly synthesized Pg-AgNPs.

The thermal analysis exhibited that independent of reaction conditions, both graphics (Figure 1A,B) exhibit the degradation of acids, alkenes, carbohydrates and aromatic compounds (esters, amino acids) highlighted by an exothermic effect around 450–550 °C. This phenomenon is noticed also on the TG-DSC curves of the dried Pg extract (Figure 1C), which confirms that the Pg extract encapsulates the pre-formed AgNPs and prevents it from oxidation and agglomeration. In addition, the formation of Pg-AgNPs was confirmed by the endothermic effect at around 960 °C without mass loss on a TG curves, highlighted on all three graphics (Figure 1A,B,D), attributed to the metallic Ag melting.

To study the ability of the capping phytocompounds from Pg extract on the surface of AgNPs, FT-IR studies were carried out. The phytochemical fingerprints of black poplar buds have depicted a plethora of phytoconstituents that are considered responsible for the therapeutic applications of this vegetable product. According to the scientific literature, black poplar buds comprise flavones (apigenin and chrysin) and flavanones (pinocembrin and pinostrombin), along with phenolic phytocompounds, including caffeic and ferulic acids, and their derivatives [60,61]. Moreover, other phytochemical investigations have shown the existence of additional natural molecules in black poplar bud extract such as tannins, oligosaccharides, triterpenes, glucose, fructose, resins and waxes. In addition, extracts also include essential oils abundant in cadinene, cineol, bisabolol, humulin, farnesol and bisabolene [62,63,64]. The FT-IR analysis of dried Pg extract (Figure 2C), extensively explained by our research team in a previous study [38], demonstrates the identification of flavonoids/flavonols, phenolic acids, phenolic glycosides and tannins. By corroborating the results from our previous study with the results of the present study, one can assume that the C=O, -C=O, C=C, =C-H and C-N functional groups were responsible for the reduction of Ag^+^ to Ag^0^. In addition, the broadening of some peaks and their intensity, on both Pg-AgNPs spectra established the capping of bioactive compounds on the surface of AgNPs. The presence of several peaks between 650–850 cm^−^^1^, which are corresponding to the stretching vibration of N-O in-plane from (NO_3_)**^−^**^1^ ion, represent another confirmation of the AgNPs formation in both samples. Our data are in agreement with the literature [48,65,66,67].

Regarding the study of the synthesized Pg-AgNPs size, shape and elemental composition, the TEM and SEM-EDX analysis revealed that the reaction conditions (temperature and/or metal salt concentration) influence the formation of newly AgNPs. Previous studies [68,69,70] demonstrates that with increase the concentration of the metal ion, increase the particle size. Another studies [71,72] sustain the fact that at higher temperature, the conversion of the metal ion to nanoparticles occurs. Regarding our study, it is possible that the concentration of AgNO_3_ salt actually determines the size and shape of the nanoparticles. Future investigations regarding the influence of each parameter on the optimization of AgNPs synthesis, with the desired shape and dimensions, are mandatory. The electron microscopy examination showed that the Pg-AgNPs_S1, obtained at 25 °C with 1 M AgNO_3_, are polydisperse, spherical/quasi-spherical with particle size between 3–60 nm; while Pg-AgNPs_S2, obtained at 60 °C with 5 M AgNO_3_, are polydisperse with irregular shape and a particle size between 5–150 nm. The EDX analysis showed that in both samples the compounds found were C, Ag, O and Cu. Our findings are similar to the results reported in the literature [73,74,75].

According to the literature, AgNPs are potential against a wide range of bacteria’s, including Gram-negative as well as Gram-positive bacteria. Due to the small size and the increased contact surface, their spreading into bacterial cells is easy. Literature reported a varied antibacterial spectrum of AgNPs including the following strains: *Streptococcus pyogens* [76], *Staphylococcus aureus* [77,78], *Bacillus subtilis* [79], *Escherichia coli* [80], *Pseudomonas aerugionosa* [81] and *Klebsiella pneumonia* [82]. In recent years, Okafor et al. [83] pointed out that the AgNPs antibacterial effect is more pronounced for *Escherichia coli* than for *Staphylococcus aureus*. The present study confirms the above-mentioned findings of the antibacterial activity of Pg-AgNPs, however, a lack of activity can be seen in the case of the Gram-negative bacteria’s, particularly *Escherichia coli* and *Pseudomonas aeruginosa*, which presented a weak inhibitory activity in case of Pg-AgNPs_S1. The antibacterial activity of AgNPs can be explained by the interaction with different cellular biomolecules such as lipids, proteins or DNA. It can be also correlated with cellular structures, which may lead to the dysfunction of bacterial cells [84]. Morones et al. [85] demonstrated the bactericidal capacity of AgNPs. Their results explained that the activity can be influenced by the size of nanoparticles (which was in the range of 10 and 100 nm) and a significant antimicrobial effect was achieved against both Gram-positive and negative bacteria. Agnihotri et al. [86] established that the antibacterial efficacy was increased when the particle sizes were low. Their study indicated that the 5 nm size AgNPs presented the fastest bactericidal activity (when it was compared with other sizes, namely 7 and 10 nm). In the present study, Pg-AgNPs exhibited decent antibacterial activity against the tested pathogens. Based on our results, the Pg-AgNPs_S1 and Pg-AgNPs_S2 were able to kill tested bacteria such as *Streptococcus pyogenes* and *Streptococcus aureus* and fungi such as *Candida species*. The antimicrobial effect of Pg-AgNPs can be related to their size or shape, therefore a larger surface area improves the interaction of Pg-AgNPs with bacterial cells. In this case, Pg-AgNPs_S2 presented a significant antimicrobial activity which can be due to their size included in the interval 5–150 nm. However, a lack of activity can be seen for Pg-AgNPs_S1 on the tested Gram-negative bacteria which can be due to their smaller size (3–60 nm) and to their cell wall structure which is different from Gram-positive bacteria. Gram-negative bacteria have a cytoplasmic membrane with a peptidoglycan layer and an outer membrane containing lipopolysaccharide [87].

Nevertheless, various aspects, including bioavailability, adverse responses, cellular interactions, biodistribution and biodegradation, must be considered in translational research. Accumulation of these NPs in the ecosystem and their absorption by living organisms might have fatal repercussions since several studies demonstrate that NPs can cause DNA and membrane damage, protein misfolding and mitochondrial dysfunction. A comprehensive toxicological assessment of NPs on plants and animals is required before they may be used in a variety of sectors [88]. Nevertheless, the mechanisms of action of AgNPs have not been fully understood until now, their structure are very complex and different, and can modulate many pathways in cancer cells. In a recent study, it has been reported that AgNPs can regulate signaling pathways or can block tumor cell metastasis by inhibiting angiogenesis [89]. Rakowski et al. [90] demonstrated that AgNPs can modulate the metastasis of MCF-7 breast cancer cells across the EMT pathway and can modify the metabolism of breast cancer cells by inducing reactive oxygen species (ROS) generation. Furthermore, Lee et al. [91] have shown that the anticancer mechanism involves also the down-regulation of the antiapoptotic proteins, such as BCL-2, and upregulation of ROS, caspase 3 and P53 proteins. Rosarin et al. [92] demonstrated the activity of green-synthetized AgNPs and highlighted that there is an association between oxidative stress, ROS generation, apoptotic morphological changes and apoptotic potential. Based on their shape and unique physical properties (such as the nanometric dimensions) AgNPs can be beneficial in developing alternative therapeutic and diagnostic strategies of cancer treatment [93]. This current work focuses on the investigation of the anticancer potential of two types of AgNPs obtained by green synthesis from ethanolic extract of *Populi gemmae*. The anticancer activity of Pg-AgNPs can be affected by physical properties, including size or shape. Compared with other shapes, spherical AgNPs exhibit better cytotoxicity due to the larger surface-to-volume ratio [94]. The synthesized Pg-AgNPs were almost spherical or quasi-spherical with sizes between 3–60 nm (Pg-AgNPs_S1) and irregular shapes (rhombohedral, triangular and spherical) with sizes between 5–150 nm (Pg-AgNPs_S2). Our results indicate that both Pg-AgNPs elicited an antiproliferative activity, however, Pg-AgNPs_S2 was more effective in decreasing breast and lung cancer cell viability than Pg-AgNPs_S1. This activity may be due to their spherical, triangular and rhomboedral shapes. Generally, AgNPs with a diameter between 10 and 100 nm can be considered suitable for anticancer treatment due to their effective delivery and permeability effects. Sizes smaller than 10 nm may undergo fast release from the normal vessels and can damage the healthy cells [95]. Consequently, based on our data, sizes between 5 and 150 nm (Pg-AgNPs_S2) can be a promising candidate for anticancer treatment. This aspect was underlined by Gomathi et al. [96] who evaluated the anticancer activity of green synthesized AgNPs (using *Tamarindus indica* fruit shell extract). The AgNPs were spherical in shape and approximately 20–52 nm in size. They have revealed that 5–120 µg/mL of AgNPs reduced the viability of MCF7 cells in a dose-dependent manner, with an IC_50_ of 20 µg/mL. Other authors have suggested that the biologically synthesized AgNPs from *Alternanthera sessilis* have significant cytotoxic activity against MCF7 breast cancer cell line. Different concentrations of AgNPs (0.1, 10 and 100 µM) showed potential anticancer activity with an IC_50_ value of 3.04 μg/mL when compared to the standard medicine used in breast cancer (Cisplatin). In accordance with the study, the higher activity can be due to the spherical shape and reduced size of particles, which was between 10 and 30 nm [97]. Another study was focused on the in vitro anticancer activity of synthesized AgNPs using pomegranate extract (*Punica granatum* L.). The AgNPs were spherical in shape, with an average size of 15.6 nm. According to the results, it was pointed out that both the extract separately, as well as the AgNPs (10–500 μg/mL), presented high toxicity against the MCF7 cancer cell line, however, the AgNPs showed higher cytotoxicity [98]. A preliminary experiment conducted by Venugopal et al. [99] determined the cytotoxic activity of AgNPs (spherical in shape with size ranging from 10 to 20 nm) against several cancer cell lines (MCF7, A549 and Hep2 cell lines). The research group has revealed that compared to the utilized aqueous extract, the viability of the selected cell lines was decreased with increasing the concentration of AgNPs (10 to 100 μg/mL) with an IC_50_ of 47.6, 48.2 and 47.1 μg/mL, respectively.

AgNPs that have sizes between 5 and 40 nm, respectively, possess predominantly spherical shapes, have been described to show cytotoxic activity through a mechanism that involves arrest in one of the phases of the cell cycle. In A549 human lung cancer cells, the nanoparticles downregulated protein-kinase C which determined cell cycle arrest at G2/M phase [100]. As previously reported in the literature, AgNPs have a significant effect in A549 lung cancer cell apoptosis. A549 cells treated with green synthesized AgNPs from *Gossypium hirsutum* (cotton) leaf extract that have a spherical shape with size range between 13 to 40 nm, induced apoptosis, achieving the cell cycle arrest in the G2/M phase. It was also pointed out that they activate the intrinsic apoptotic pathways, decreasing the Bcl-2 and Bax genes [101]. Another paper confirmed that the biosynthesized AgNPs with a spherical arrangement in shape and size of 10 to 20 nm, have been very effective against A549 cells in a concentration-dependent manner. Increasing the concentration, the A549 cell viability was decreased, and an inhibition percentage of 94% was achieved at 80 µg/mL concentration [102].

Medicinal plants are frequently considered to be of lower risk compared to synthetic drugs [103]. Regarding the vegetal product *Populus nigra* L. buds, it is generally considered to be safe, but due to the phytochemical composition, administration may be limited in some cases. It is important to mention that patients with allergies/sensibility to salicylate derivatives, or pregnant women and those who are breastfeeding should avoid the use of products containing black poplar [104]. At the same time, there are some drugs that can cause interactions; therefore, it should avoid the concomitant administration of black poplar extract together with NSAIDs, different anticoagulants, hypoglicemiants and antihy-pertensives [105,106]. NPs, in general, can quickly be accumulated in different human organs, such as the liver, kidney, spleen, even heart or brain, and may cause a potential toxic effect [107,108]. Recently it was described that there are some specific factors that can mediate the mechanism of their toxicity, among which can be listed the generation of ROS, necrosis, apoptosis and inflammation, however, these pathways need to be acknowledged better [109]. In vitro toxicity studies suggested that AgNPs can be toxic to the lung, liver, brain and reproductive organs cells, however, the in vivo toxicity action needs to be elucidated, as AgNPs showed significant toxicity in case of inhalation, ingestion or intravenous administration [110,111]. Nevertheless, there are some important factors that are responsible for their toxic potential, such as the size, shape, dose and surface area of the NPs [112]. The toxicity of metallic nanoparticles may also depend on different environmental conditions, solubility, oxidation state or ligands [95,113]. Accordingly, as was already indicated in this work, the green synthesis of AgNPs using different plant extracts can be assessed as a replacement for the chemical and physical synthesis methods used at this time. Until now, many researchers interpreted and used various vegetal products, such as leaves, barks, fruits, roots and seeds, for the synthesis of NPs [114,115]. Given the fact that, green synthesis is environment friendly and having no toxicity effects, this approach opens a new era of safe nanotechnology [95].

To the best of our knowledge, no prior studies have examined the in vitro antibacterial and antiproliferative effect on selected cell lines of the green synthesized nanoparticles obtained from *Populus nigra* L. buds. Consequently, our findings seem to be promising since the use of AgNPs is considered an effective anticancer approach, however, upcoming investigations of these preliminary reports are necessary to investigate the mechanism of action.

## 5. Conclusions

The current study describes the distinguished design of AgNPs by green synthesis using an ethanolic extract of *Populi gemmae*. The physicochemical analysis showed that the obtained Pg-AgNPs are stable, polydisperse and small in size, suitable for biomedical applications. It was demonstrated that the size and shape of nanoparticles can be adjusted by controlling the reaction temperature and metal salt concentration. The results have shown that the newly Pg-AgNPs exhibited a good antibacterial activity on Gram-positive bacteria as well as on fungi, Pg-AgNPs_S2 being more reactive. A good antibacterial activity is associated with a good particle size distribution, due to the high interaction between the cell membrane of bacteria or fungi and AgNPs. In the set experimental conditions, Pg-AgNPs provoked, in a dose-dependent manner, an antiproliferative potential against the selected cancer cell lines. The Pg-AgNPs_S2 (obtained at 60 °C, using AgNO_3_ of 5 M) presented a stronger antiproliferative activity on both studied cancer cell lines. Additional investigations need to be performed that include in vivo analysis in order to have a full snapshot of the possible therapeutic potentials and benefits in the management of different cancers.

## Figures and Tables

**Table 1:** Antimicrobial activity of Pg-AgNPs_S1.

Microbial Strains	Inhibition Diameters (mm)	MIC (mg/mL)	MBC or MFC (mg/mL)
*Streptococcus pyogenes*	17	25	25
*Staphylococcus aureus*	16	50	50
*Escherichia coli*	10	-	-
*Pseudomonas aeruginosa*	9	-	-
*Candida albicans*	17	25	25
*Candida parapsilosis*	16	25	25

**Table 2:** Antimicrobial activity of Pg-AgNPs_S2.

Microbial Strains	Inhibition Diameters (mm)	MIC (mg/mL)	MBC or MFC (mg/mL)
*Streptococcus pyogenes*	21	12.5	12.5
*Staphylococcus aureus*	20	12.5	25
*Escherichia coli*	17	25	25
*Pseudomonas aeruginosa*	16	25	25
*Candida albicans*	18	12.5	25
*Candida parapsilosis*	17	12.5	25

## Data Availability

Not applicable.
